# COVID-19-Induced Vestibular Neuritis, Hemi-Facial Spasms and Raynaud's Phenomenon: A Case Report

**DOI:** 10.7759/cureus.11752

**Published:** 2020-11-28

**Authors:** Rachana Vanaparthy, Srikrishna V Malayala, Mamtha Balla

**Affiliations:** 1 Pulmonology, Oregon Health & Science University, Portland, USA; 2 Internal Medicine, Temple University Hospital, Philadelphia, USA; 3 Internal Medicine, ProMedica Toledo Hospital, Toledo, USA

**Keywords:** covid-19, vestibular neuritis, hemifacial spasms, neurological manifestations, raynaud’s phenomenon, cutaneous manifestations, urticaria.

## Abstract

The coronavirus disease 2019 (COVID-19) pandemic has created a global health crisis. Though respiratory symptoms have been the usual manifestations, the presentation in some cases may be atypical with various neurological and cutaneous manifestations. We present a case of a 63-year-old female diagnosed with COVID-19 and associated rare manifestations during her visit to Europe.

## Introduction

The outbreak of coronavirus disease 2019 (COVID-19), caused by severe acute respiratory syndrome coronavirus 2 (SARS-CoV-2), was first identified in Wuhan, a city in Hubei province in China in December 2019. As of September 21, 2020, nearly 31.1 million cases and 962,000 deaths were reported in 213 countries and territories. Severe acute respiratory syndrome CoV (SARS-CoV) and novel CoV were believed to share the same receptor, angiotensin-converting enzyme (ACE), hence the virus was termed SARS-CoV-2, and World Health Organization named the disease as coronavirus disease 2019 (COVID-19) in February 2020 and declared it as a pandemic on March 11, 2020 [1]. 

Initially, fever, cough, shortness of breath, and myalgia were reported as common symptoms and pneumonia-like features in chest computerized tomography (CT) scan in patients affected by COVID-19 [1]. But, later, various neurological manifestations were noticed. Olfactory and gustatory involvement resulting in anosmia and dysgeusia are common neurological symptoms in mild cases. Guillain-Barre syndrome and inflammation of the brain, spinal cord, meninges, cranial nerve, and peripheral nerve involvement are reported [2]. Various cutaneous manifestations like a morbilliform rash, urticaria, vesicular eruptions, acral lesions, petechiae, chilblains, livedo racemosa, and distal necrosis are also seen [3].

We report a 63-year-old Caucasian female who had a diagnosis of COVID-19 with other associated manifestations during her visit to a small country in Europe. 

## Case presentation

A 63-year-old Caucasian female with a past medical history of aplastic anemia, mitral valve prolapse with regurgitation, celiac disease, and motion sickness presented with a runny nose and breathlessness. She did not report fever, chills, cough, or chest pain. Given the past history of aplastic anemia, she took over-the-counter iron pills for shortness of breath with no improvement. She is a resident of the United States but an avid traveler and was in Europe when the symptoms developed. The symptoms developed in March 2020 when there were no reported COVID-19 cases. 

When there were no signs of improvement after a few days of symptomatic management, the nasal polymerase chain reaction (PCR) for the SARS-CoV-2 test was performed in an ambulatory setting, which turned out to be positive, confirming the diagnosis of COVID-19, and she was advised home quarantine.

**Figure 1:**
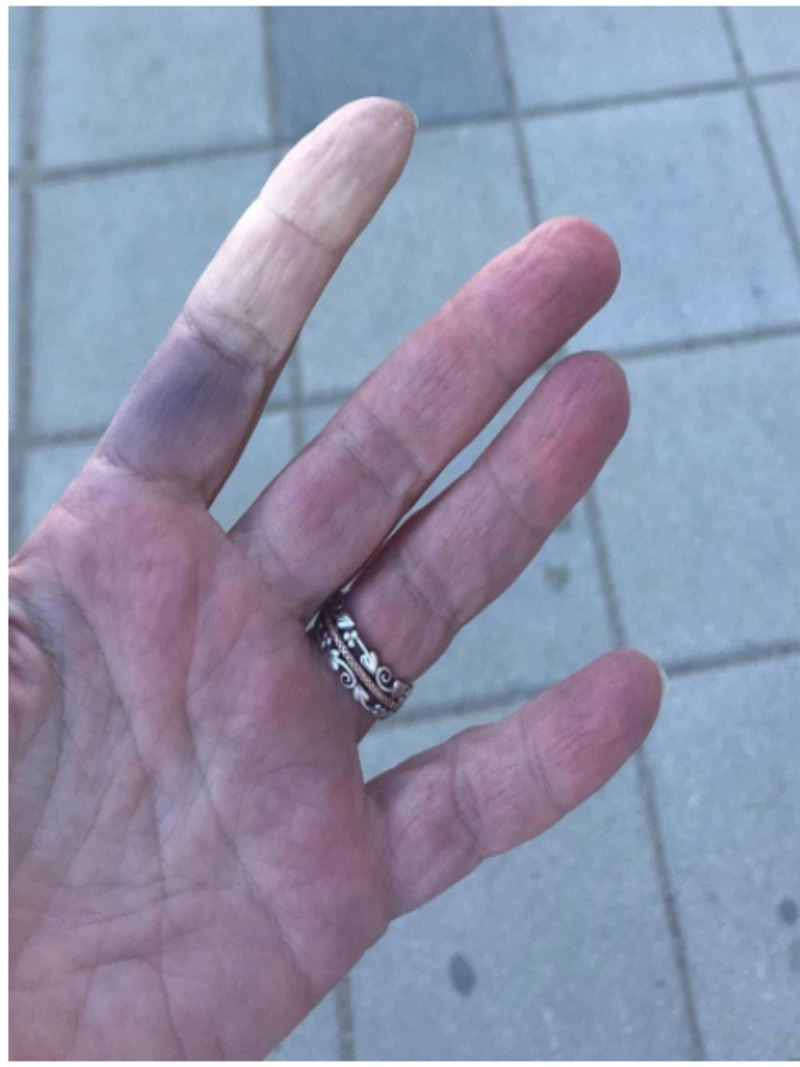
Raynaud's phenomenon after COVID-19 infection

About four weeks after the initial episode, the patient developed twitching of the left eye and left cheek, diarrhea, generalized weakness, palpitations, sleep disturbances, decreased appetite, skin rash, anosmia, and dysgeusia. Twitching was involuntary, initially started near the left eye, and progressed to the left side of the face. It was not associated with pain, loss of sensations, or numbness. There were eight to 10 painful, red skin lesions around 3 mm in size in the lower face, especially around the mouth. A clinical diagnosis of herpes labialis caused by herpes simplex type-1 (HSV-1) was made. There was no associated itching, bleeding, blistering, or discharge. She also noticed purple discoloration at the base and whitish discoloration at her fingertips with temperature changes, as shown in Figure 1. She continued conservative management with ample hydration, antipyretics, and over-the-counter aspirin, multivitamins, and calcium supplements. Over the next four weeks, she slowly had some clinical improvement. Subsequently, she was tested for COVID-19 every week until she was negative on the 58th day.

One week after she tested negative for COVID-19, she suddenly developed chills and vomitings. She woke up in the middle of the night with dizziness, a sense of the room spinning and an unsteady gait. She did not have tinnitus or hearing loss.

She immediately took medical attention and the physical examination showed a strong nystagmus to the right. Dix-Hallpike maneuver was performed, and she was confirmed with vertigo and diagnosed with post-viral vestibular neuritis. Initially, she was treated with meclizine, antiemetics, and Cawthorne vestibular rehabilitation exercises. Her symptoms did not improve with symptomatic management and subsequently she was given 60 mg prednisone tapered gradually over the next 10 days. On the 10th day of prednisone, the patient noticed a sudden onset of flashes and floaters in the left eye. A slit-lamp examination diagnosed posterior vitreous detachment (PVD) of the left eye. PVD was attributed to an increase in intra-ocular pressure with the use of steroids. There was a slight improvement in her vision after cessation of steroids. 

Several weeks later, she developed high-grade fever, arthralgias, arthritis, and a non-itchy urticarial rash all over her chest and abdomen eight hours following the intake of 2 g amoxicillin/clavulanic acid for a dental procedure. Fever was as high as 102F, not associated with chills and rigors. She did not report lymphadenopathy or pedal edema. Symptoms subsided in a couple of days without any treatment. Five months after her positive COVID-19 test, she was tested for COVID-19 immunoglobulin G (IgG) antibodies and found to be negative. 

## Discussion

As we are learning and trying understand the COVID-19 disease, an emerging infection that resulted in a global pandemic, we are well aware that respiratory symptoms are most distinctively seen in COVID-19 patients. It is believed that a pro-inflammatory cytokine release, known as ‘cytokine storm,’ causes pulmonary damage by COVID-19. This is also the likely mechanism of central nervous system (CNS) pathology related to COVID-19. ACE2 is the functional receptor for the SARS-CoV-2 virus, present in multiple organs such as the lungs, heart, nervous system, skeletal muscles, blood vessels, kidney, liver, and gastrointestinal tract. The ACE2 receptor is found remarkably in the epithelial lining of lung alveoli, enterocytes of the small intestine, the endothelial lining of arteries and veins, and arterial smooth muscles of all organs [4]. Hence, COVID-19 can affect any of the organs, as mentioned above.

SARS-CoV-2 enters the CNS through either a hematogenous or retrograde neuronal route; the neural invasion mechanism is mainly through the cribriform plate, olfactory nerve, thalamus or brainstem thus resulting in the suppression of central cardiorespiratory drive [5]. Mao et al., in a study in Wuhan published in April 2020, mentioned that the neurological manifestations are often seen in critically ill patients [6]. Later, it was noticed that many patients with few or no symptoms of COVID-19 disease also had neurological symptoms [7]. Further, Mao et al. classified neurological manifestations as dizziness, headache, impaired consciousness, ataxia, seizure, and acute cerebrovascular disease into central nervous system manifestations. Nerve pain, taste, smell, and vision impairment into peripheral nervous system manifestations and skeletal muscular injury manifestations [6].

Our patient presented with features of peripheral nerve involvement (anosmia and dysgeusia), cranial nerve involvement (hemifacial spasms and vestibular neuritis), and Raynaud’s phenomenon (RP). Peri- and post-infectious anosmia and dysgeusia could be secondary to olfactory nerve lesions from viral infection. The majority of patients presented at the same time or a few days after the onset of other COVID-19 symptoms [8]. Though anosmia is often the presenting symptom in many COVID-19 patients, our patient had this symptom a few weeks after the initial presentation. 

Hemifacial spasm is a neuromuscular movement disorder with slight intermittent contractions or twitching of the muscles innervated by the facial nerve (cranial nerve VII). Though twitching is irregular initially, it may be severe, more persistent, and can spread to the muscles of facial expression in the following months. Sometimes, a mild peripheral weakness can develop. The spasms are due to compression of the facial nerve by the artery at the root exit of the brainstem. The clinical features are essential in diagnosing the disease; electromyography and magnetic resonance imaging (MRI) are additional diagnostic modalities to determine the underlying cause [9]. Few case reports described the association of bilateral facial paralysis with paresthesia, a subtype of Guillain- Barre syndrome, and COVID-19 [10]. Few studies describe facial nerve involvement, causing Bell’s palsy but relatively do not know much about the hemifacial spasms in COVID-19. 

Acute vestibular neuritis (VN), or peripheral vestibulopathy (PVP), is defined as a lesion of the eighth cranial nerve’s vestibular component without auditory deficits. It is a clinical entity with the features of vertigo or dizziness along with nausea or vomiting, gait instability, head motion intolerance, and nystagmus, which is developed over minutes or hours. The most common cause is the reactivation of latent herpes simplex virus, especially herpes simplex virus type 1 (HSV-1) [11]. In some cases, it was noted that COVID-19 infection could cause vestibular neuritis in susceptible populations [12].

The possible source of vestibular neuritis in our patient is post-viral inflammation of the vestibular nerve, probably due to COVID-19 infection or perhaps a combination of COVID-19 and HSV infections. Given that VN’s features were seen after a week of negative COVID-19 test, we suspect COVID-19 as the more common reason. Like any other case of vestibular neuritis, symptomatic treatment with antiemetics, antihistamines, anti-cholinergic agents, anti-dopaminergic agents, and benzodiazepines are given [13]. Often, corticosteroids show significant improvement of peripheral vestibular function recovery in severe cases [14]. Resumption of regular activity and vestibular rehabilitation with Cawthorne Cooksey exercises promotes central vestibular compensation [15]. 

COVID-19 has also been associated with various cutaneous manifestations like a morbilliform rash, urticaria, vesicular eruptions, acral lesions, petechiae, chilblains, livedo racemosa, and distal necrosis [3,16]. The possible mechanism for these cutaneous manifestations is the lymphocytic vasculitis induced by blood immune complexes activated by cytokines [17]. A few case reports noted that cutaneous findings were present before the respiratory symptoms developed. In contrast, a few case reports found that these findings are seen several days after the onset of symptoms [16]. Our patient presented with RP during the disease and urticarial rash several weeks later despite no previous history. Kolivras et al. described the first case of COVID-19 induced chilblains due to the delayed expression of the interferon (IFN)-inducible genes, further exacerbating hypercytokinemia [18]. Chilblains are an inflammatory skin reaction to an abnormal vascular response to cold. They present as tender, pruritic, red lesions on the dorsal aspect of fingers or toes [19]. In contrast, RP is the triphasic color change with initial pallor, followed by cyanosis and erythema due to abnormal vasoconstriction of the digital arterioles when exposed to cold. RP’s diagnosis is mainly made with history and it predominantly affects the hands. It usually responds well with general measures like smoking cessation, avoidance of cold exposure, repeated trauma, and vasoconstricting drugs. The few patients who do not respond to general measures require pharmacological treatment that includes a calcium channel blocker, losartan, and an angiotensin II receptor blocker [20]. The RP manifestations in our patient improved with simple conservative measures and did not require any pharmacological management as such.

## Conclusions

Our case report mentions the neurological and cutaneous manifestations in a patient with COVID-19 with an excellent prognosis. To our knowledge, this is the first case report of COVID-19-induced hemifacial spasms and Raynaud’s phenomenon. Until further validation by subsequent studies, we like to alert the clinicians to various disease presentations in COVID-19, who are immediately tested and treated. Though fever and respiratory symptoms remain the hallmark of early identification and management of COVID-19; the treatment should not be delayed while keeping in mind the other manifestations. 
